# Supplementary figures and images for: Acute inhibition of centriolar satellite function and positioning reveals their functions at the primary cilium
Source: PLoS Biol. 2020 Jun 18;18(6):e3000679. doi: 10.1371/journal.pbio.3000679 (PMC7326281; doi:10.1371/journal.pbio.3000679)

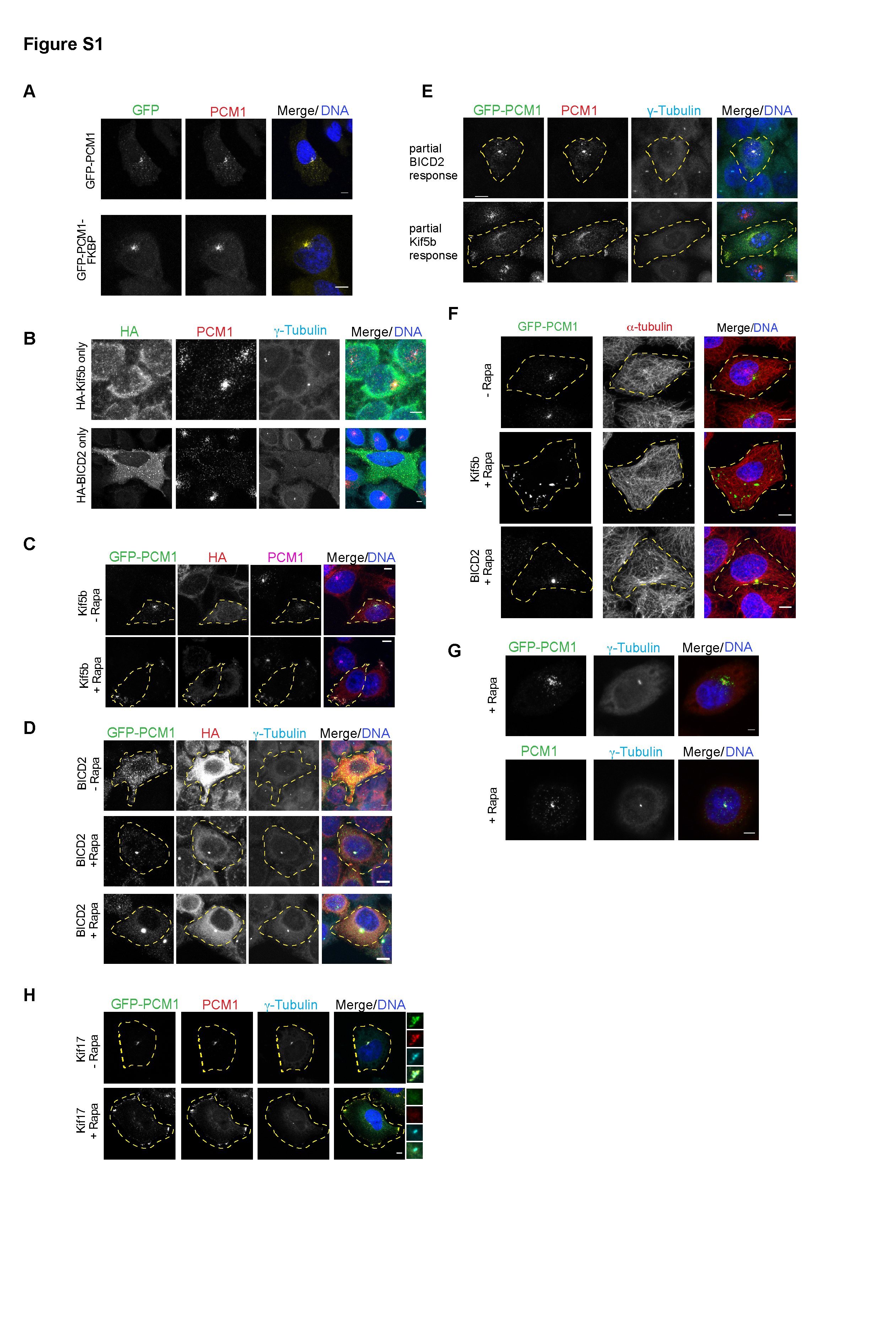

Supplement: S1 Fig — (A) Localization of GFP-PCM1 and GFP-PCM1-FKBP in cells. HeLa cells were transfected with GFP-PCM1 or GFP-PCM1-FKBP, fixed after 24 hours, and stained for GFP, PCM1, and DAPI. (B) Effects of HA-BICD2-FRB or HA-Kif5b-FRB expression on satellite distribution. HeLa cells were transfected with HA-BICD2-FRB or HA-Kif5b-fRB, fixed after 24 hours, and stained for HA, PCM1, gamma-tubulin, and DAPI. (C, D) Validation of GFP-PCM1-FKBP and endogenous PCM1 mispositioning upon rapamycin-induced dimerization in cells. Hela cells co-expressing GFP-PCM1-FKBP with (C) HA-Kif5b-FRB or (D) HA-BICD2-FRB were treated with rapamycin for 1 hour, fixed 24 hours after transfection, and stained for GFP, HA, PCM1, and DAPI. Cells that were not treated with rapamycin were processed in parallel as controls. (E) Representation of partial distribution of satellites upon rapamycin induction. HeLa cells co-expressing GFP-PCM1-FKBP with HA-Kif5b-FRB or HA-BICD2-FRB were treated with rapamycin for 1 hour, fixed 24 hours after transfection, and stained for GFP, HA, PCM1, and DAPI. Partial distribution was defined by GFP-PCM1-FKBP signal in the pericentrosomal area in Kif5b-expressing cells and signal in the region excluding the centrosomal area in BICD2-expressing cells. (F) Expression of GFP-PCM1-FKBP with HA-Kif5b-FRB or HA-BICD2-FRB and their redistribution upon rapamycin induction do not perturb the microtubule network. Cells were stained for GFP, alpha-tubulin, and DAPI. (G) Rapamycin treatment did not perturb satellite distribution in wild-type cells and cells expressing only GFP-PCM1-FKBP. Cells were treated with rapamycin for 1 hour, fixed after 24 hours, and stained for GFP or PCM1, gamma-tubulin, and DAPI. (H) Co-expression of GFP-PCM1-FKBP with the constitutively active HA-Kif17 (1–181 aa)-FRB targets satellites to the cell periphery, where satellite clusters are heterogeneously distributed. Transfected HeLa cells were treated with rapamycin for 1 hour, fixed after 24 hours, and stained for [file pbio.3000679.s001.tif]

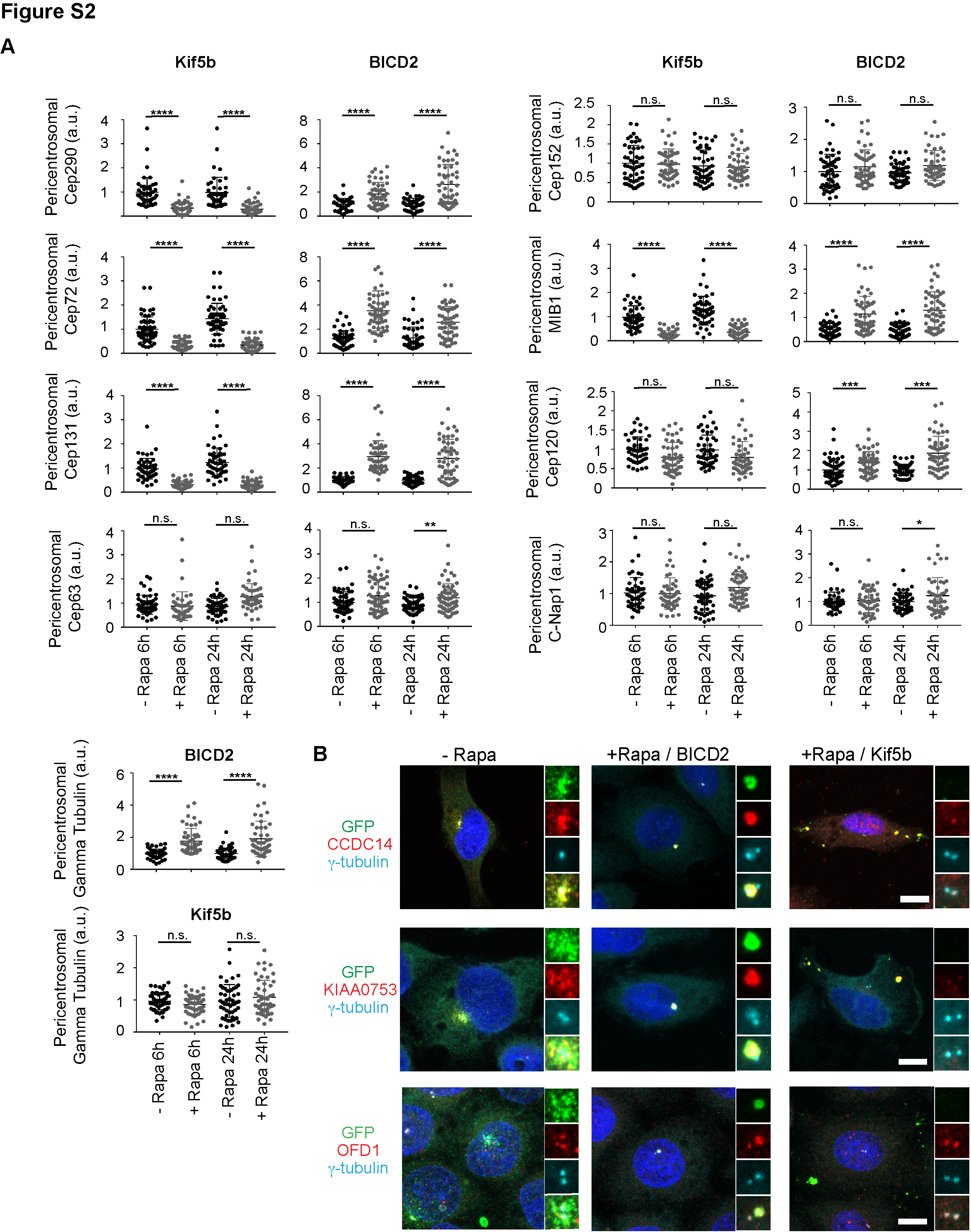

Supplement: S2 Fig — (A) HeLa cells co-expressing GFP-PCM1-FKBP with HA-Kif5b-FRB or HA-BICD2-FRB were treated with rapamycin for 1 hour followed by fixation at 6 and 24 hours. Cells that were not treated with rapamycin and exhibited pericentrosomal clustering of GFP-PCM1-FKBP–like endogenous PCM1 of wild-type cells were processed in parallel with controls. Cells were stained with antibodies anti-GFP to identify cells with complete redistribution to the cell periphery or center, anti–gamma-tubulin to mark the centrosome, and antibodies against the indicated proteins. Fluorescence intensity at the centrosome was quantified and average means of the levels in control cells were normalized to 1. n ≥ 25 cells per experiment. Data represent the mean value from two experiments per condition ± SD (**p < 0.01, ***p < 0.001, ****p < 0.0001, n.s. nonsignificant). Error bars = SD. Source data can be found in S3 Data. (B) Control and rapamycin-treated cells were stained for GFP, gamma-tubulin, and indicated satellite proteins. Images represent centrosomes in cells from the same coverslip taken with the same camera settings. DNA was stained with DAPI. Cell edges are outlined. Scale bars, 10 μm; all insets show 4× enlarged centrosomes. BICD2, bicaudal D homolog 2; FKBP, FK506 binding protein 12; FRB, FKBP12-rapamycin-binding; GFP, green fluorescent protein; HA, hemagglutinin; Kif5b, kinesin family member 5b; PCM1, pericentriolar material 1 (TIF) [file pbio.3000679.s002.tif]

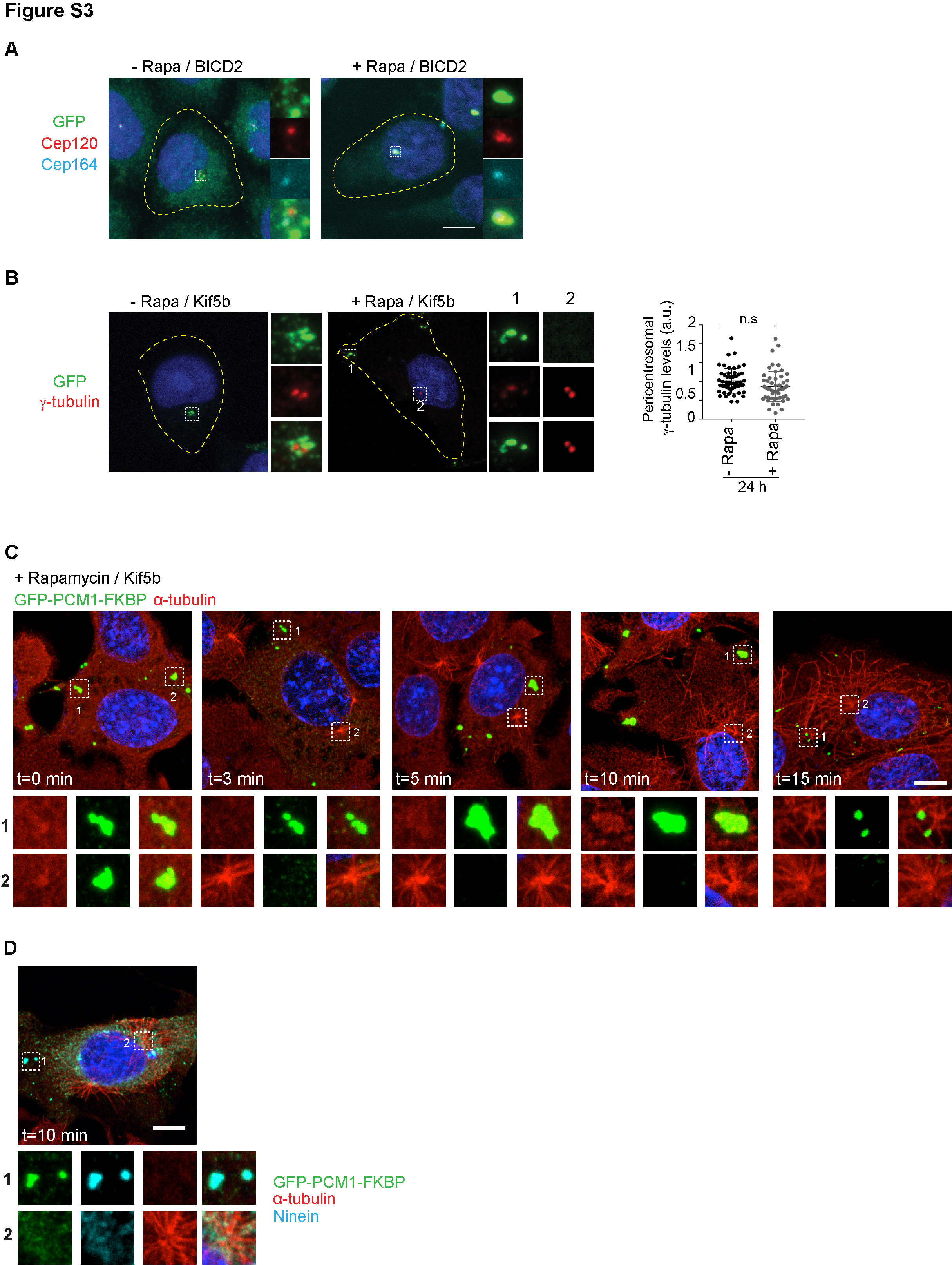

Supplement: S3 Fig — (A) The daughter centriole protein Cep120 was redistributed to the mother centriole in BICD2-expresing cells with centrosomal satellite accumulation. HeLa cells co-expressing GFP-PCM1-FKBP with HA-BICD2-FRB were treated with rapamycin for 1 hour, fixed at 24 hours, and stained for GFP, Cep120, Cep164, and DAPI. Cells that were not treated with rapamycin were used as a control. (B) Gamma-tubulin localization in control cells and in Kif5b-expressing cells with peripheral satellite clustering. HeLa cells co-expressing GFP-PCM1-FKBP with HA-Kif5b-FRB were treated with rapamycin for 1 hour, fixed at 24 hours, and stained for GFP, gamma-tubulin, and DAPI. Images represent centrosomes in cells from the same coverslip taken with the same camera settings. Cells that were not treated with rapamycin were processed in parallel as a control. Fluorescence intensity at the centrosome was quantified, and average mean of the levels in control cells were normalized to 1. n ≥ 25 cells per experiment. Data represent mean value from two experiments per condition ± SD (n.s. nonsignificant, ****p < 0.0001). Error bars = SD. Source data can be found in S3 Data. (C) Effect of gamma-tubulin accumulation at the peripheral satellites on microtubule nucleation. Rapamycin-treated IMCD3peripheral cells were treated with DMSO or 10 μg/mL nocodazole for 1 hour. After microtubule depolymerization, cells were washed, incubated with complete media for the indicated times, fixed, and stained for GFP, alpha-tubulin, and DAPI. (C) Rapamycin-treated IMCD3peripheral cells were treated with DMSO or 10 μg/mL nocodazole for 1 hour. After microtubule depolymerization, cells were washed, fixed 10 minutes after nocodozole washout, and stained for GFP, alpha-tubulin, ninein, and DAPI. Scale bars, 10 μm; all insets show 3× enlarged centrosomes. BICD2, bicaudal D homolog 2; FKBP, FK506 binding protein 12; FRB, FKBP12-rapamycin-binding; GFP, green fluorescent protein; HA, hemagglutinin; Kif5b, kinesin family member [file pbio.3000679.s003.tif]

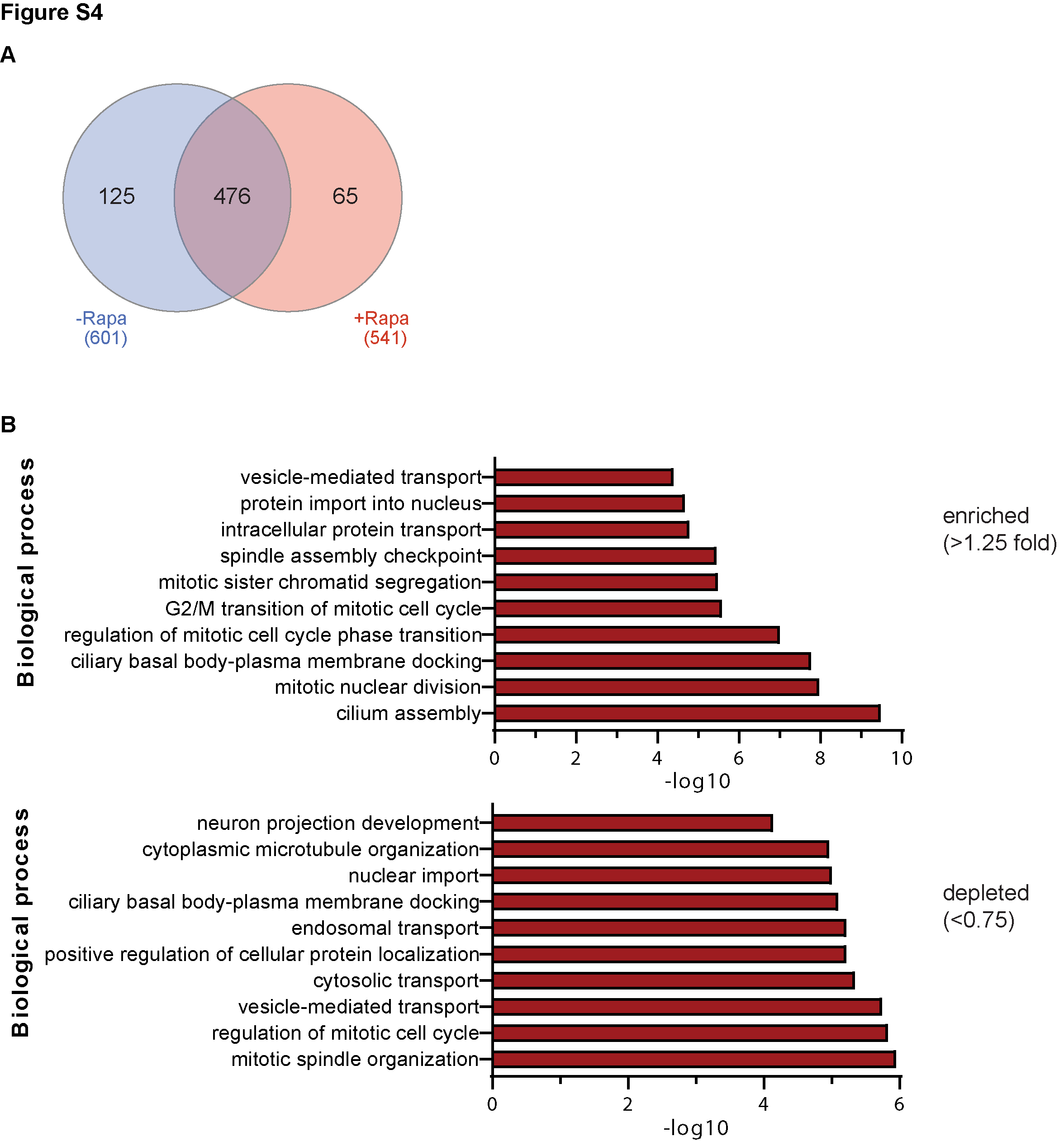

Supplement: S4 Fig — (A) The control and peripheral PCM1 interactomes share 476 components. A total of 65 proteins were specific to peripheral satellites and 125 proteins were specific to control cells. (B) GO-enrichment analysis of the proteins enriched (>1.25-fold relative to control) and depleted (<0.75-fold relative to control) in cells with peripheral satellite clustering after rapamycin treatment based on their biological processes. The x-axis represents the log-transformed p-value (Fisher’s exact test) of GO terms. Source data can be found in S4 Data. GO, Gene Ontology; PCM1, pericentriolar material 1 (TIF) [file pbio.3000679.s004.tif]

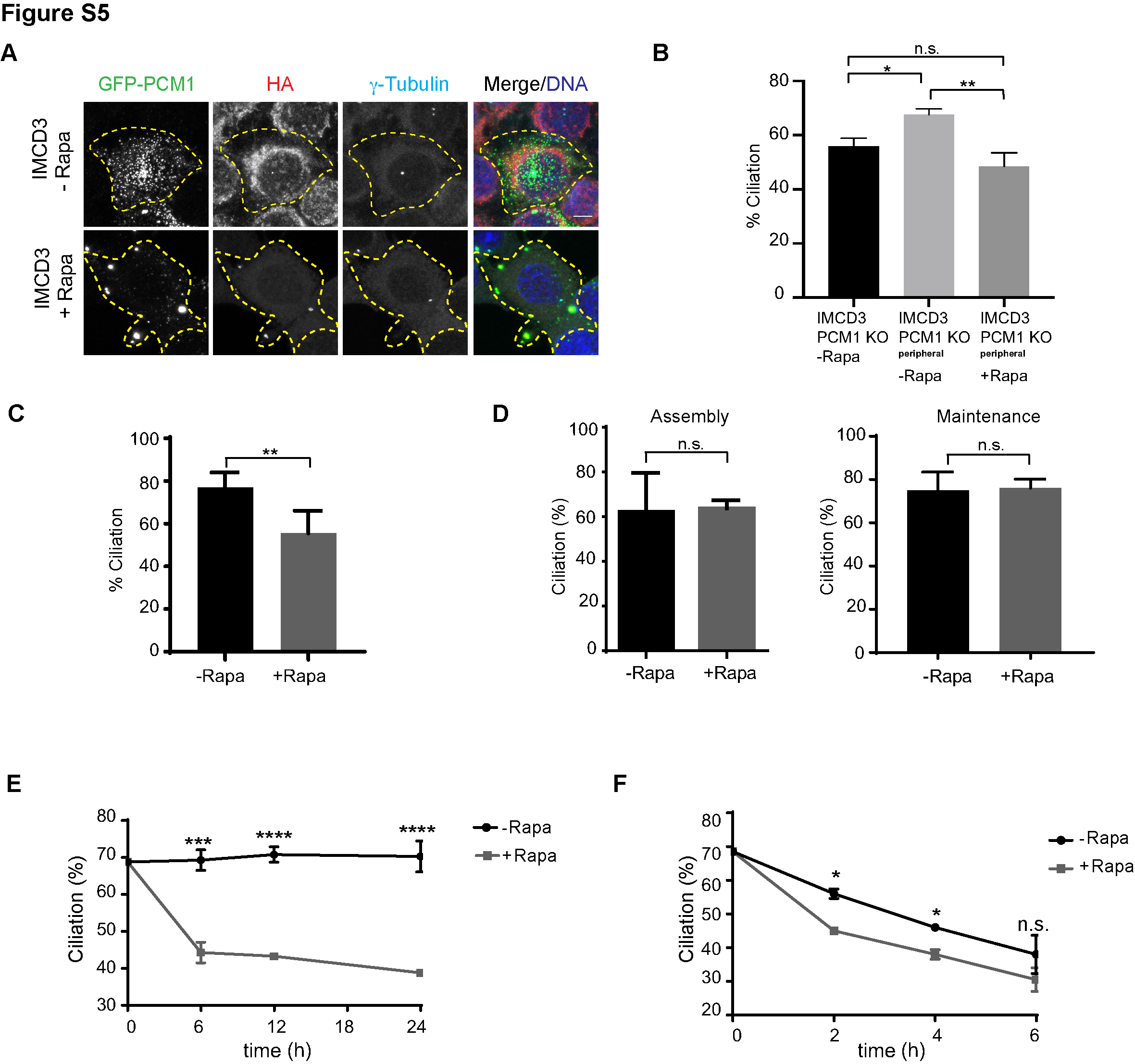

Supplement: S5 Fig — (A) Validation of IMCD3peripheral cells for expression of HA-Kif5b and GFP-PCM1-FKBP. Control and rapamycin-induced IMCD3peripheral cells were stained for GFP, HA, gamma-tubulin, and DAPI. (B) GFP-PCM1-FKBP restores ciliogenesis defects of satellite-less IMCD3 PCM1 KO cells and peripheral satellite clustering compromises their ciliogenesis efficiency. IMCD3 PCM1 KOperipheral cells stably expressing GFP-PCM1-FKBP and HA-Kif5b-FRB (−Rapa and +Rapa) and IMCD3 PCM1 KO cells were serum-starved for 48 hours, and the percentage of ciliated cells was determined by staining for acetylated-tubulin. Results shown are the mean of three independent experiments ± SD (= 50 cells/experiment, *p < 0.05, **p < 0.01). (C) Quantification of the percentage of ciliogenesis in control and rapamycin-treated IMCD3peripheral cells 48 hours after serum starvation. Results shown are the mean of two independent experiments ± SD (= 50 cells/experiment, **p < 0.01). (D) Rapamycin treatment by itself in IMCD3 cells does not affect the efficiency of ciliogenesis and cilium maintenance. The experiments were performed in IMCD3 cells following the experimental outline for ciliogenesis and maintenance experiments in Fig 4, and the fraction of ciliated cells were quantified after 48 hours of serum starvation for the cilium assembly experiment and 24 hours after rapamycin treatment for maintenance experiments. Results shown are the mean of two independent experiments ± SD (= 200 cells/experiment). (E) Effect of peripheral satellite clustering on cilium maintenance. IMCD3peripheral cells were serum-starved for 48 hours, treated with rapamycin for 1 hour, and the percentage of ciliated cells was determined over 24 hours by staining for acetylated tubulin. Cells that were not treated with rapamycin were used as a control. Data represent the mean value from two experiments per condition ± SD (***p < 0.001, ****p < 0.0001). (F) Effect of peripheral satellite clustering on cilium disassembly. IMCD3peripheral w [file pbio.3000679.s005.tif]

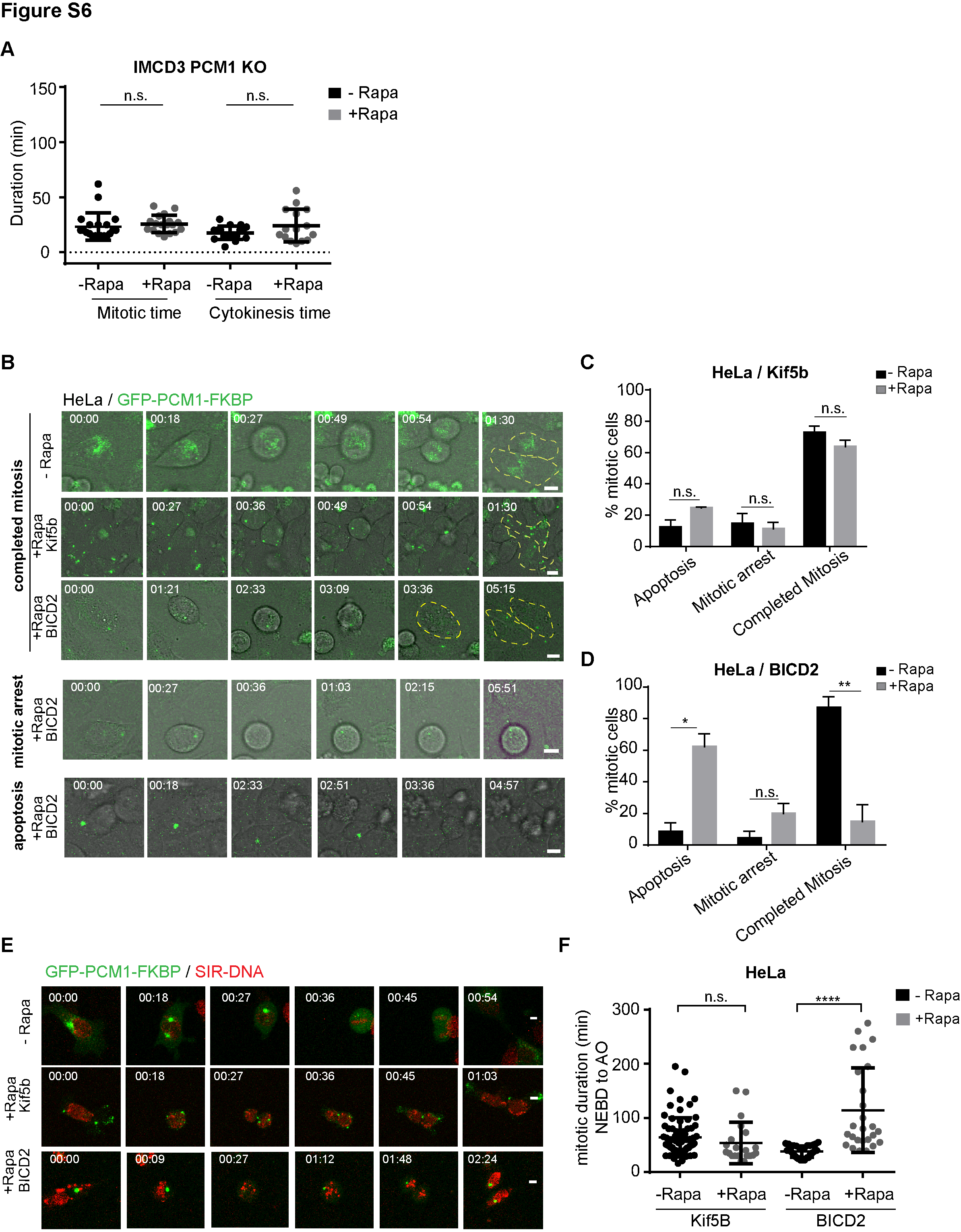

Supplement: S6 Fig — (A) Rapamycin treatment itself does not affect mitotic time and cytokinesis time of IMCD3 PCM1 KO cells. Control and rapamycin-treated IMCD3 PCM1 KO cells were imaged over 16 hours using time-lapse imaging. Mitotic and cytokinesis times were quantified. Data represent mean value from two experiments per condition ± SD. (B) Effect of peripheral and centrosomal satellite clustering on mitotic dynamics of satellites and mitotic progression of HeLa cells. GFP-PCM1-FKBP was co-transfected to HeLa cells with HA-Kif5b-FRB or HA-BICD2-FRB, treated with rapamycin, and imaged every 4.5 minutes for 16 hours. Representative brightfield and GFP-PCM1-FKBP fluorescence still frames from time-lapse experiments for the different phenotypes quantified were shown. Cell edges are outlined. Scale bar, 10 μm. (C, D) Quantification of (B). Percentage of control, and rapamycin-treated mitotic cells were imaged by the time-lapse imaging and videos were analyzed to classify mitotic cells into categories of cells that completed mitosis, cells that underwent apoptosis, and cells arrested in mitosis for more than 5 hours. n > 20 cells per experiment. Data represent mean ± SD of three independent experiments. (E) Effect of peripheral and centrosomal satellite clustering on mitotic time in HeLa cells co-expressing GFP-PCM1-FKBP with HA-Kif5b-FRB or HA-BICD2-FRAB. Control and rapamycin-treated cells were stained with SIR-DNA and imaged by time-lapse microscopy. (F) Quantification of (E). n > 20 mitotic cells per experiment. Data represent mean ± SD of two independent experiments. Error bars = SD. Source data can be found in S6 Data. BICD2, bicaudal D homolog 2; FKBP, FK506 binding protein 12; FRB, FKBP12-rapamycin-binding; GFP, green fluorescent protein; HA, hemagglutinin; IMCD3, inner medullary collecting duct; Kif5b, kinesin family member 5b; KO, knockout; n.s., non-significant; PCM1, pericentriolar material 1; SIR, silicone rhodamine (TIF) [file pbio.3000679.s006.tif]
