# Supplementary material for: Acute inhibition of centriolar satellite function and positioning reveals their functions at the primary cilium
Source: PLoS Biol. 2020 Jun 18;18(6):e3000679. doi: 10.1371/journal.pbio.3000679 (PMC7326281; doi:10.1371/journal.pbio.3000679)
Supplement: S1 Raw Images — The unprocessed western blot data for Fig 3B. BioID, Biotin Identification; FKBP, FK506 binding protein 12; PCM1, pericentriolar material 1 (PDF) [file pbio.3000679.s020.pdf]

Raw Western Blot Data for Fig. 3B

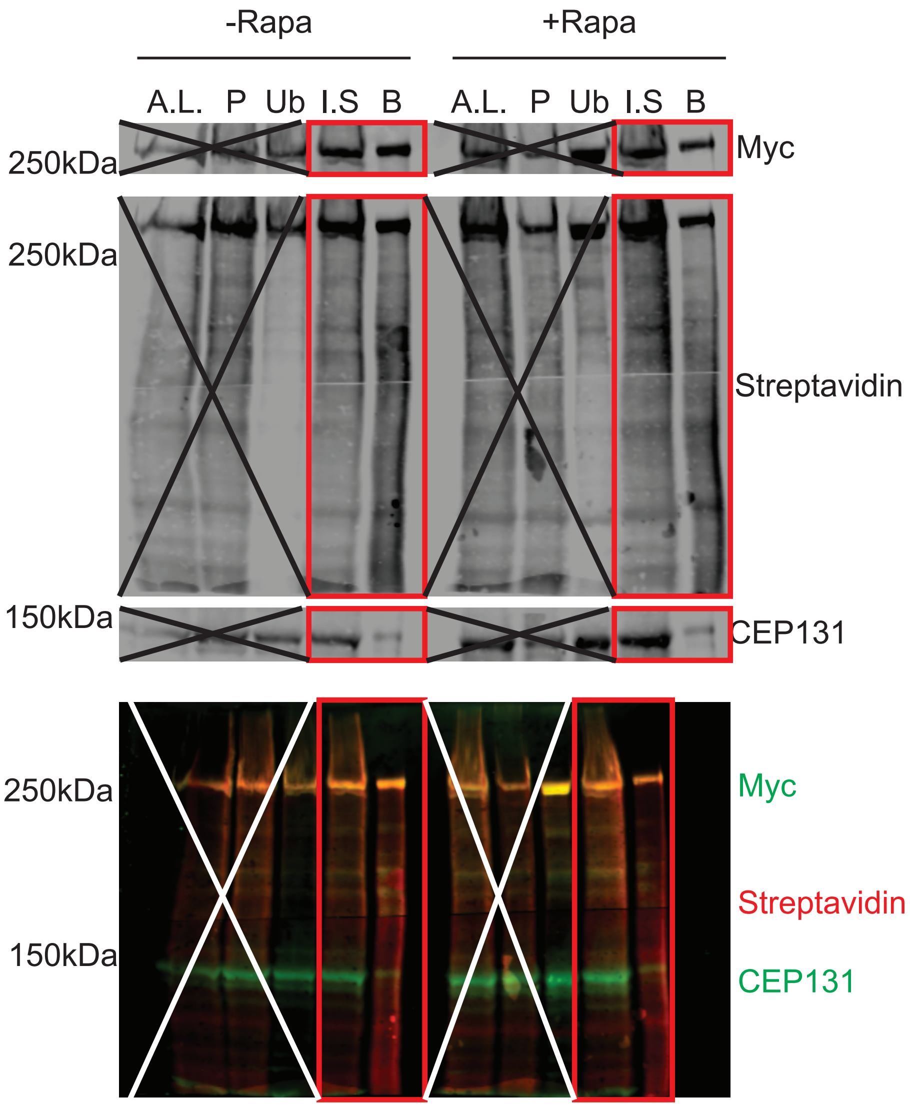

A.T: After lysis
P: Insoluble pellet
Ub: Unbound
I.S: Initial sample
B: Beads

Whole membrane was first blotted with Streptavidin in Red then membrane was cut between 150kDa and 250kDa.
Top part of the membrane was blotted with Myc in green and bottom half of the membrane was blotted with CEP131 in green
