# Supplementary material for: Acute inhibition of centriolar satellite function and positioning reveals their functions at the primary cilium
Source: PLoS Biol. 2020 Jun 18;18(6):e3000679. doi: 10.1371/journal.pbio.3000679 (PMC7326281; doi:10.1371/journal.pbio.3000679)
Supplement: Attachment — Submitted filename: PointByPointResponse_final.pdf [file pbio.3000679.s022.pdf]

MS #: PBIOLOGY-D-20-00224
Title: Acute inhibition of centriolar satellite function and positioning reveals their functions at the primary cilium

We appreciate the time and effort the reviewers took to thoroughly review our manuscript and thank them for their overall positive assessment and constructive feedback. We believe that we have addressed all the reviewer points by performing new experiments and rephrasing the relevant parts of the manuscript accordingly as well as by expanding discussion on functions and mechanisms as revealed by application of satellite trafficking assays in cells.

Reviewer#1:
The manuscript by Aydin et al. deals with the fascinating and contentious issue of the function of centriolar satellites. They developed a clever rapamycin inducible approach to translocate satellites either to the cell periphery or to the centrosome. Using these tools they perform BioID mass spec analysis to try and determine the components of the satellites away from the potentially contaminating centrosome. Using this approach they found many of the known satellite proteins but also identified some that may warrant a deeper look based on the differential enrichment. Finally and most significantly they utilize these tools to address a more nuanced function for satellites and identify 2 novel functions in cilia maintenance and disassembly. I think this is an interesting paper that has identified some important and novel functions for satellites. However there a couple of experiments that could potentially really strengthen the paper and are worth considering.

Comments:
1- *My primary suggestion to improve this paper would be to perform the functional experiments with the BICD2-PCM1 constructs. The authors state "In contrast to HA-Kif5b, we noticed that HA-BICD2 expression by itself resulted in satellite dispersal throughout the cytosol (Fig. S1B), which is consistent with its previous characterization as a dominant negative mutant that impairs dynein-dynactin function [24-26]. Therefore, we did not use BICD2-based centrosomal satellite clustering as a way to assay satellite functions in ciliated and proliferating cells." However, I feel that an opportunity has been missed. While I agree that the DN dispersal phenotype complicates things, I still think that there is something to be gained from the forced enrichment of PCM1 at the centrosome (which is considerable). Does this enrichment disperse in a cell cycle dependent manner (e.g. Fig 5A) or is it forced to maintain its centrosomal localization and if so to what end. Similarly, one could predict that this enrichment might enhance ciliogenesis or maintenance above WT levels or slow disassembly. This analysis feels lacking from the data.*

- The reviewer raises a very good point. In fact, we initially planned to perform functional assays in cells with satellite clustering both at the centrosome and cell periphery. We hypothesized two possible outcomes from this comparative analysis 1) antagonistic phenotypes if there is an inverse correlation between satellite proximity to centrosomes and centrosome/cilium-mediated functions, 2) similar phenotypes if irreversible satellite peripheral and centrosomal clustering and disruption of their physiological distribution interferes with satellite functions . Although we successfully generated IMCD3 and IMCD3 PCM1 KO cells stably co-expressing HA-Kif5b-FRB with GFP-PCM1-FKBP, we were not able to generate cell lines that stably expressed HA-BICD2-FRB by itself or with GFP-PCM1-FKBP, likely due to constitutive inhibition of dynein activity. We now included in pg. 15 the following sentence to clarify why we did not investigate the phenotypic consequences of centrosomal satellite clustering in all functional experiments:

"We were unable to generate cell stably expressing GFP-PCM1-FKBP and HA-BICD2-FRB, likely due to the lethality of constitutive BICD2 expression and subsequent inhibition of dynein activity."

- Given that we could not generate HA-BICD2-FRB-expressing stable lines, we employed transient transfection of HeLa cells for comparative analysis of centrosomal and peripheral satellite clustering. As

the reviewer pointed out, HeLa cells co-expressing GFP-PCM1-FBKP with BICD2-FRB had very strong centrosomal clustering of satellites, validating our approach (Fig. 1D, Fig. S1D). Here is the summary of our results and their interpretation:

a) In Fig. 2 and Fig. S2, we used these cells to quantitatively compare the pericentrosomal levels of centrosome proteins previously identified as satellite residents. For a subset of these proteins including Cep131, Cep290, Cep72 and MIB1, the centrosomal and peripheral satellite clustering had antagonistic effects. Interestingly, for another subset of proteins that include Cep120, Centrin, Cep63 and gamma-tubulin, the centrosomal clustering increased their pericentrosomal levels and the peripheral clustering had no effect. This difference might be due to more efficient targeting of cargoes by satellites, which physiologically already clusters pericentrosomally. Collectively, these results show that the function of satellites in pericentrosomal protein targeting varies depending on the specific centrosome cargo.

b) In the revised manuscript, we performed new experiments to assay the mitotic phenotypes associated with satellite clustering at the periphery or the centrosome. 24 h after transfection, we treated cells with rapamycin and examined mitotic satellite dynamics and mitotic phenotypes using immunofluorescence and time-lapse imaging experiments.
   - Analogous to the behavior of peripheral satellite clusters of Kif5b-expressing IMCD3 and HeLa cells cells, the centrosomal satellite clusters of BICD2-expressing cells did not dissolve. The centrosomal clusters remained associated with either one spindle pole or two spindle poles. This unequal distribution of clusters between spindle poles might be due to their strong association mother centrioles of interphase cells. Together, these experiments show that clustering of satellites at the periphery and the centrosome inhibits their mitotic dissolution. We included these results in Fig. 5A of the revised manuscript.
   - Analogous to IMCD3^{peripheral} cells, HeLa cells with peripheral satellites clusters did not have defects in mitotic progression and mitotic time (Fig. 6B-F). However, HeLa cells with centrosomal satellite clusters had a significant increase in mitotic defects that included prolonged mitosis and increased apoptosis (Fig. S6B-F). These results together identify functions for proper satellite positioning during mitotic progression. Given that satellite dissolution during mitosis is inhibited both in cells with centrosomal and peripheral satellites, we can conclude that these phenotypes are not consequences of defects in mitotic satellite remodeling and that centrosomal clustering of satellites specifically interferes with mitosis either due to defects in protein targeting or microtubule organization/dynamics.

c) We were not able to perform phenotypic characterization of cilium-related phenotypes upon transient transfection of IMCD3 cells due to two reasons. In our experience, transfection by itself interferes with efficient ciliogenesis. Even transfection of GFP compromises ciliation to some extent. Second, the timeline of the cilium disassembly and maintenance experiments is not compatible with the expression of proteins upon transient expression. Therefore, we cannot conclude on how centrosomal satellite clustering affects cilium biogenesis.

In addition to adding these new results to the revised manuscript, we included their discussion in the related respective parts of the "Discussion" section.

2- *In Figure S3C it is hard to make the firm conclusion that the ectopic satellites are not acting as an MTOC. This is due to the large amount of MTs after 15min, I think doing it at 5 min would provide more useful information. Or perhaps showing the lack of a MT - end protein such as ninein.*

- For the revised manuscript, we repeated the microtubule regrowth experiments in IMCD3 PCM1^{peripheral} cells. This time, we fixed the cells at more frequent intervals after nocodazole washout (0/3/5/8/10/15 min), which allowed us to assay microtubule nucleation with higher temporal resolution. We included representative images for cells stained for microtubules and GFP-PCM1-FKBP at the

indicated time points in Fig. S3C. In particular, the cells fixed 3 and 5 min after washout convincingly showed that the gamma-tubulin positive satellite clusters did not nucleate microtubules while centrosomes did, as shown in Fig. S3C. These new results confirm our initial conclusions.

- As suggested by the reviewer, we also stained cells fixed at different time points after nocodazole washout with ninein, which at the centrioles localizes to subdistal appendages and the proximal end of centrioles. Additionally, ninein was one of the first proteins shown to be regulated by satellites (Dammermann, A and Merdes, A. JCB 2002) and also was identified as a satellite component in recently published satellite proteome (Quarantotti et al. EMBO J 2019). In agreement with its satellite interaction, we found that ninein localizes to peripheral satellite clusters, yet these clusters did not nucleate microtubules, as shown in Fig. S3D. Although we were not able to stain cells with more specific microtubule minus end proteins such as CAMSAPs or Patronin, these results strongly demonstrate lack of microtubule nucleation at peripheral clusters.

- We note that recent satellite proteomics studies identified HAUS complex components and gamma-tubulin as part of satellites and yet satellite do not function as acentrosomal MTOCS. Together with our results, we suggest that peripheral satellite clusters or satellites lack the modifications or regulatory proteins essential for microtubule nucleation. We discuss this point with the following sentence in pg. 12:
"Thus, the concentration of gamma-tubulin at the peripheral satellites does not assign them MTOC activity, which might be due to lack of modifications or regulatory proteins required for microtubule nucleation."

Minor:
1- *The description of Cep164 localization as a ring like localization seems misleading. I suspect that it is likely spherical but that given a the imaging plane looks ring like.*

We agree with the reviewer. We revised this sentence in pg. 9 as follows:
"Staining of BICD2-expressing cells for satellites and the mother centriole protein Cep164 revealed tight clustering of satellites around the mother centriole."

2- *"Cep120 was strongly enriched in the mother centriole in BICD2-expressing cells (Fig. 3A)." Should be Fig. S3A.*
We corrected this mistake.

3- *On Page 10 Figure 3D should be 3E.*
We corrected this mistake.

4- *Error bars on Figure S5A.*

We revised this figure to include data from three experimental replicates and plotted the data with statistical analysis in Fig. S5A. The statistical analysis confirmed that GFP-PCM1-FKBP restores ciliogenesis phenotypes of IMCD3 PCM1 KO cells.

5- *I found these sentences confusing: "While 70.9% ± 3.3 of control cells were ciliated, only 52.8% ± 0.9 of IMCD3 PCM1 KOperipheral cells were ciliated (p<0.01) (Fig. 4C). Similarly, only 77.35% ± 1.65% IMCDperipheral cells ciliated relative to 54.4% ± 1.4 control ciliating population (p<0.01) (Fig. 4D)." please clarify.*

We revised this sentence as follows to clarify it: "While control cells ciliated at 70.9% ± 3.3, IMCD3 PCM1 $KO^{peripheral}$ cells ciliated at 52.8% ± 0.9 ($p<0.01$) (Fig. S5B). ……… Similarly, $IMCD^{peripheral}$ cells had a significant reduction in their ciliation efficiency relative to control cells (control: 77.35% ± 1.65%, $IMCD3^{peripheral}$: 54.4% ± 1.4) ($p<0.01$) (Fig. 4C)."

***6-** The figures need to be cleaned up with proper scale bars and such. 5A cells do not seem to be at the same scale.*
We rechecked the scaled bars throughout the manuscript, included the missing ones and corrected the ones the scale bars in Fig. 5A.

***7-** The movies were not properly referenced in the text.*
We corrected the referencing of movies 1/2/3/4 in the text.

Reviewer 2: Philippe Bastin – please note that this reviewer has waived anonymity
This manuscript addresses the role of the cellular positioning of centrosome satellites in their function. For this, the authors use an elegant approach to conditionally reposition the central satellite protein PCM1 to the cell periphery (use of kinesin) or the cell centre (use of dynein). After a clear and well-documented description of the system, two major findings came up:

(1) Some centrosomal proteins are "trapped" with PCM1 and have limited/no access to the centrosome while others are not affected, indicated two possible pathways for centrosomal targeting. This is supported by immunofluorescence assays and proteomic studies with proximity labelling.

(2) The system is used to investigate convincingly the contribution of PCM1 and centriolar satellites in the maintenance and the disassembly of the primary cilium whereas inhibition of satellite dispersion during mitosis does not seem to cause mitotic defects

Overall, it is a nice piece of work that is well executed and well written. The authors also discuss the limitations of their system (for example satellite dispersion upon HA-BICD2 expression). The message of the paper will be of interest for cell biologists interested in organelle function and positioning, an important theme in developmental and cellular biology. One might say that the results were somehow expected since structures matter where they are but this reviewer believes in data and getting experimental evidence is therefore essential. The data reported are quite compelling, although they could be further discussed. I have several suggestions that I hope will contribute to improve the manuscript. Most things can be addressed without further experiments (or by including data that are possibly available), although some experiments could bring useful information (see below). In my view, reviewing is a dialogue and I will be happy to read the response of the authors.

***1.** The discussion is a bit superficial and focuses a lot on future work, it somehow looks more similar to a grant application. This reviewer believes that the authors have nice and exciting data that deserve more exhaustive discussion. Here are a few thoughts: centrosomal proteins seem to fall in two categories: those that rely on PCM1 and those that don't. What is the relationship between proteins belonging to each of this group? Are they known to interact? What about the conservation profile? PCM1 is not present in all eukaryotic species. Does its presence somehow correlate with the presence of proteins that rely on satellite for centrosomal targeting? Is there a relationship with proteins associated to cilia or only found at the centrosome level?*

We agree with the reviewer that the discussion section of our manuscript should be more inclusive of our results and their interpretations in addition to future directions. We thank the reviewer for suggesting questions that deserves further discussion in light of our data. By taking into account suggestions for further discussion and the new results included in revised manuscript, we expanded the discussion section in the following areas:

- We determined the phenotypic consequences of satellite clustering at the centrosomes or periphery in HeLa cells. In contrast to no mitotic defects we found in IMCD3 and HeLa cells with peripheral clusters, HeLa cells with centrosomal satellite clusters had a significant increase in mitotic defects that included prolonged mitosis and increased apoptosis (Fig. 6B-F). These results together identify functions for proper satellite positioning during mitotic progression. Given that satellite dissolution during mitosis is

inhibited both in cells with centrosomal and peripheral satellites, we can conclude that these phenotypes are not consequences of defects in mitotic satellite remodeling and that centrosomal clustering of satellites specifically interferes with mitosis either due to defects in protein targeting or microtubule organization/dynamics.

- We examined the dynamics of ciliogenesis and the cilium length in IMCD3 PCM1 $KO^{peripheral}$ cells, which revealed defective ciliation at time points after 6h post-serum starvation and a significant defect in cilium length. Together with the results of cilium maintenance/disassembly and proteomics experiments, we further discussed the mechanisms by which satellites take part in cilium assembly and maintenance, with a particular focus on regulation of IFT-B complex.

- To determine the set of proteins regulated by satellites at the level of centrosomal/ciliary protein targeting, we took a targeted approach (quantitative immunofluorescence) and proteomics approach (BioID). For the targeted approach, we determined the pericentrosomal levels of multiple centrosome proteins implicated in a range of functions and have different subcentrosomal localizations (Table in Fig. 2B). The behavior of these proteins did not tightly correlate with their functions and localizations as summarized below in their classification into different groups:
<u>1st group:</u> Their pericentrosomal levels were antagonistically regulated in cells with centrosomal and peripheral clusters: Cep290, Cep72, Cep131, MIB1, OFD1, CCDC14, KIAA0753
<u>2nd group:</u> Their pericentrosomal levels increased in cells with centrosomal clusters, did not change in cells with peripheral clusters: Centrin, CEP120, CEP63, gamma-tubulin, C-Nap1
<u>3rd group:</u> Their pericentrosomal levels did not change in both cases: CEP152

Here are our answers to the specific questions that the reviewer raised:
1- *What is the relationship between proteins belonging to each of this group? Are they known to interact?*
- The proteins regulated by PCM1 did not have functional clustering. They are implicated in functions ranging from centriole duplication (KIAA0753, CCDC14) to ciliogenesis (CEP290, CEP72, OFD1, CEP131). Lack of functional clustering also is apparent for proteins that are not regulated by PCM1. The GO-enrichment analysis of the PCM1 proximity interactome at the periphery confirms lack of functional clustering, but also reveals enrichment/depletion of proteins implicated ciliogenesis and mitosis.
- As for relationship at the interaction level, there are subcomplexes of proteins that were shown to physically interact and are also regulated by PCM1. For example, CCDC14 and KIAA0753 interact and regulate centriole duplication. Moreover, Cep290 and Cep72 interact and regulate ciliogenesis. This results might suggest function of satellite in assembling subcomplexes and/or regulating centrosomal targeting of subcomplexes.

2- What about the conservation profile? PCM1 is not present in all eukaryotic species. Does its presence somehow correlate with the presence of proteins that rely on satellite for centrosomal targeting?
A subset of PCM1-regulated proteins including Cep72, CCDC14, KIAA0753 have similar pattern of evolutionary conservation to PCM1. However, other PCM1-regulated proteins including Cep290, Cep131 and centrin are conserved in organisms that lack PCM1. Therefore, there is not a correlation between evolutionary conservation and satellite-mediated regulation.

2- Is there a relationship with proteins associated to cilia or only found at the centrosome level?
We only tested centrosome proteins in the targeted quantitative immunofluorescence approach, therefore we cannot use this data to comment on this question. However, the proteins enriched or depleted in PCM1 peripheral proximity maps showed that PCM1 regulates a wide range of centrosome proteins, some of which also have ciliary pools (IFT complex components, BBSome complex). We have not found PCM1 interactors that localizes only to cilia, but not centrosomes. These results suggests that satellite might mediate its functions at the cilia through centrosomal targeting of their residents.

Together with work by us and others on satellites, we acknowledge that these results still still does not address the mechanisms and the rules underlying the selectivity of satellites for their residents at the level of regulatory mechanisms. We aim to investigate this significant, but unresolved question in future studies.

2. *I was a bit surprised to see that cilium length was not reported (although the use of ARL13b/acetylated tubulin for measurements is mentioned in M&M). Possibly the authors have the data. The reason why it is not mentioned might be found in a recent publication of the same group (Odabasi et al EMBO19) that showed that deletion of PCM1 reduces the number of cilia but not the length of remaining cilia in IMCD3 cells. In any case, the length of the remaining cilia should be discussed here. It is possible that the phenotype would be more pronounced if it was taken into account. I agree it sounds unlikely given the KO results but as said above, evidence is better. Nevertheless, it would not be the first time that a mutant/modification of a protein (in this case its location) induces a stronger phenotype than a deletion. If cilium length is unaffected, that means we are in an "all or nothing" situation. Either cells manage to do a cilium of normal length, either they don't and in that case. How do the authors interpret this? This is worth discussing.*

We thank the reviewer for proposing an experiment we should have performed to corroborate our conclusions on cilium assembly and discussing potential mechanisms for phenotypic differences between acute and chronic inhibition of satellites. For the revised manuscript, we quantified cilium length from three independent experiments using acetylated tubulin/Arl13b staining and included this data in Fig. 4C. This analysis showed that the length of cilia in IMCD3 PCM1 KO^{peripheral} cells with peripheral satellites were slightly (about 14%) but significantly shorter than the control cells (-Rapa: 2.9 μm ± 0.1, +Rapa: 2.5 μm ± 0.1, $p<0.01$). As the reviewer discussed, we did not find a similar cilium length defect in IMCD3 PCM1 KO^{peripheral} cells, which indicated that satellite-less cells were in an “all or nothing” situation as suggested by the reviewer. Additionally, the cilium length phenotype also shows that acute inhibition of satellites through their misdistribution results in a stronger phenotype than their constitutive loss. Taken together with the cilium maintenance defects (Fig. 4E) and enrichment of IFT components in peripheral satellite clusters (Fig. 3E), the underlying mechanism for the cilium length phenotype might be the defective centrosome and/or ciliary recruitment of the IFT-B complex. Unfortunately, the IFT antibodies we tried did not work in IMCD3 cells and therefore, we could not test this hypothesis. Based on the above findings and the new data we included, we now also expanded the discussion on the link between satellites and cilium biogenesis.

3. *This reviewer likes the proteomic data but is a bit amazed that some of the results are not experimentally validated/investigated a bit further. For example, the presence of many members of the intraflagellar transport B (IFTB) complex is remarkable and could suggest a depletion of these factors essential for cilium elongation. This could help answering the question raised above. In case enough IFTB proteins are available, the cilium might elongate. If not, IFT trafficking might be insufficient to sustain ciliogenesis initiation. This could be relevant in case the cilium forms like in Chlamydomonas where most of the IFT material is injected at once at the onset of ciliogenesis (see papers of Wallace Marshall MBoC2005; JCB2009). I could not find the information for IMCD cilium assembly though. Immuofluorescence with anti-IFT antibodies in conditions where PCM1 and satellites are sent to the periphery could be very useful in proving (or not) this hypothesis. I know that the quality of anti-IFT antibodies in mammalian cells has been an issue for the field, but I will be happy to hear the opinion of the authors.*

We thank the reviewer for his comments on the possible mechanisms by which satellites might regulate cilium assembly and maintenance through the IFT-B complex. The defects in cilium assembly, maintenance and length together with the identification of IFT-B components in PCM1 proximity maps strongly suggest that there is a tight molecular and functional relationship between satellites and IFT-B complex. We currently do not know the nature of this relationship. It could be at the level of IFT-B complex assembly, centrosomal and ciliary targeting and/or ciliary trafficking. To test this, we first

aimed to test whether there are defects in the recruitment of IFT-B components to the basal body and the cilium in cells with peripheral satellite clusters. As the reviewer acknowledged, the antibodies we ordered for the available IFT-B components (IFT88 and IFT81) did not work in immunofluorescence experiments in IMCD3 cells. In fact, this is a common problem we experience with mouse cell lines in particular. The antibodies against multiple centrosome/cilium proteins that work for immunofluroecence in human cell lines do not work in mouse cell lines.

In our Odabasi et al. EMBO Reports 2019 study, we successfully used the rabbit polyclonal IFT88 antibody (Proteintech, Cat#13967-1-AP) to quantify its basal body and ciliary levels. However, we ran out of this antibody and the new batch we ordered did not work despite our efforts of optimizing fixation/permeabilization/blocking conditions. We also ordered the IFT81 antibody (Proteintech, Cat#11744-1-AP), which again worked in staining cilia in RPE1 cells but not IMCD3 cells. Therefore, we were not able to test the hypothesis that satellites mediate its functions at the cilia in part through regulating IFT-B recruitment to the basal body or the cilia using the satellite trafficking assay. We plan to investigate the evident relationship in future studies after developing more tools (i.e. stable lines).

We included further discussion on the relationship between satellites and the IFT-B complex in the revised manuscript.

***4.** Finally, the authors did an excellent job for the investigation of cilium maintenance and disassembly with several data points (Fig. 4E&F). By contrast, only one time point is shown for the assembly (48h, Fig. 4B). At that stage, cilia are likely to have reached mature length. This is unfortunate because following how cilia grow with intermediate time points would give really nice insights on the way they rely on PCM1 proper location for construction. I don't know if the authors have done this experiment already (it's possibly another story!) but I would advise them to have a try.*

The reviewer suggests a great experiment that allowed us to investigate the link between satellites and cilium assembly dynamics. For the revised manuscript, IMCD3 PCM1 $KO^{peripheral}$ cells were treated with rapamycin and serum-starved for 2h, 6 h, 16 h, 24 h and 48 h followed by fixation and staining with cilium markers. We plotted the percentage of ciliated cells at different time points for control and rapamycin-treated cells in Fig. 4D. While there was no significant difference in the fraction of ciliated cells at 0h, 2 h and 6 h; ciliation efficiency in rapamycin-treated cells were significantly lower than control cells at 16 h, 24 h and 48 h. These results suggest that satellite proximity around the basal body is not required for initiation of ciliogenesis.

To determine the molecular events of ciliogenesis associated with these different timepoints after serum starvation, we did an extensive literature search. As the reviewer acknowledged in his comment#2, despite the lack of better characterization of the timing of IFT-B complex recruitment and activity during ciliogenesis, these events remain poorly understood in cells that use the extracellular ciliogenesis pathway. One study that examined dynamics of ciliogenesis relative to different ciliary markers is the Wu et al. NCB 2018 paper, which showed identified Myosin-Va-mediated preciliary vesicle transportation to the mother centriole as the first stage of ciliogenesis. In IMCD3 cells, they detected formation of MyoVa-associated PCVs/CVs at 15 min and 30 min post-serum starvation and elongating axoneme at 1 h and 6 h post-serum starvation. Additionally, in our Odabasi et al. EMBO Reports 2019 study, we showed that the satellite-less IMCD3 cells proceeded normally through early steps of mother centriole maturation (i.e. removal of CP110, recruitment of transition zone proteins), but they were defective in the recruitment of IFT88 to the basal body. Collectively, these results provide further support that satellites might regulate ciliogenesis at the level of regulating the IFT-B complex.

Based on the above findings and the new data we included, we now revised the discussion section related to the link between satellites and cilium biogenesis

Reviewer#3:
This manuscript documents the development of methods based on rapamycin-induced heterodimerization of FKBP and FRB to redistribute pericentriolar satellites from their normal positions, loosely dispersed around the centrosome, either to clusters at the periphery of the cell (minus the centrosome) or to a single large cluster focused on the mother centriole. The methods are validated in great detail. The authors then use immunofluorescence microscopy to analyze how select satellite proteins change in abundance when the satellites are repositioned. Using BioID, they next determine that the satellite interactome remains relatively unaltered when the satellites are redistributed to peripheral clusters. The authors then carry out studies to determine how ciliary assembly, maintenance, and disassembly are affected by repositioning of the satellites to the cell periphery. The results provide the first direct evidence that proximity of the satellites to the cilium's base is important for primary cilium maintenance and disassembly. In addition to extending the general importance of cell organelle positioning for function of the cell organelle, the results raise interesting questions for future research. In general, the manuscript is scholarly, well written, and scientifically sound; it is appropriate for publication in PLOS Biology. However, it would be improved if the following issues were addressed prior to publication.

P. 4: *The major section that begins here is called "Results and Discussion." There also is a "Discussion" section that begins on p. 14. The section beginning on p. 4 probably should be simply "Results."*
We corrected the "Results and Discussion" section as the "Results" section in the revised manuscript.

P. 5: *The phrase "co-expressing the FKBP-fusion of satellites as the cargo of interest" is awkward and unclear.*
We revised this sentence as "…by co-expressing FKBP-fusion of satellites with FRB-fusion of constitutively active plus-end or minus-end directed motor domains".

P. 5: *HA-BICD2 expression by itself caused satellite dispersal, presumably as a result of its previously documented dominant-negative effect on dynein-dynactin function. Here or later, briefly explain why addition of rapamycin to these cells nevertheless results in clustering of satellites at the centrosome, presumably due to dynein moving on microtubules to their minus ends.*

The mechanism by which N-terminal fragment of BICD2 behaves as a dominant-negative mutant and causes satellite dispersal is likely through outcompeting endogenous activating adaptors that mediate satellite interactions with the dynein-dynactin complex. Although the dynein/dynactin adaptor(s) for satellites is not known, the published satellite proteomes suggest Hook3, BICD2 and ninein as putative adaptors. Rapamycin addition induces chemical dimerization of BICD2-N with satellites. Given the ability of BICD2-N itself interacts with dynein, this forced interaction must be sufficient to couple satellites to dynein and induce their movement to microtubule minus ends, as confirmed by efficient centrosomal clustering of satellites (Fig. 1D). We now included the following conclusion and discussion in pg. 7:

"In contrast to HA-Kif5b, we noticed that HA-BICD2 expression by itself resulted in satellite dispersal throughout the cytosol (Fig. S1B), likely through outcompeting endogenous activating adaptors that couple satellites to dynein-dynactin complex. Of note, this is consistent with its previous characterization as a dominant-negative mutant that impairs dynein-dynactin function [24-26]. Although the activating adaptors required for minus-end-directed movement of satellites is not known, the chemical dimerization of BICD2 with satellites is sufficient to induce their centrosomal clustering in our assay (Fig. 1D)."

***P. 7:** Clustering of satellites is dependent on microtubules, but the clusters do not disperse when microtubules are depolymerized. Briefly discuss the likely reason that the clusters remain intact in the absence of microtubules.*

The reviewer raises a very good discussion point that pertains to the material state of satellites. Previous work by us and others showed that satellites have liquid-like properties like fusion, splitting and dissolution (Conkar et al. 2019 Scientific Reports, Rai et al. Nature 2018). The irreversible aggregation of satellite clusters at the periphery can thus be explained by fusion of satellites into solid-like aggregates when forced into close proximity. In agreement, the peripheral and centrosomal satellite clusters also did not dissolve during mitosis (Fig. 5A). Notably, in unperturbed cells, satellites do not fuse into these aggregates and are kept in the proximity of the centrosome, suggesting that there are mechanisms that inhibit their aggregation. In preliminary FRAP experiments in half and full aggregates, we showed that the signal did not recover in the bleached region, which confirms that aggregates do not arrange internally. We discussed these points in two different parts of the manuscript.

- Results section in pg. 9:
  "The clusters remained mostly intact and did not split into smaller granules upon depolymerization of microtubules by nocodazole treatment, suggesting that satellite granules fuse into stable solid-like clusters when forced into close proximity."
- Discussion section in pg. 25:
  "Notably, satellite granules fuse and form stable aggregates when forced into close proximity at the cell center or periphery [14], suggesting that cells have mechanisms to inhibit aggregation of satellites. Future studies are required to address several key questions that pertains to satellite mechanisms and cellular distribution: What is the precise mechanism that establishes and maintains pericentrosomal satellite clustering and inhibit their aggregation?"

***P. 8:** "except for centrin." I could not find data for centrin. Add "data not shown"?*
We thank the reviewer for pointing out the lack of the representative images/quantification for centrin in rapamycin-treated cells. In the revised Fig. 2C (last panel), we included representative images for localization of "centrin" in control and rapamycin-treated Kif5b- or BICD2-expressing cells along with quantification of its pericentrosomal levels. As we discussed in the manuscript pg. 11, these results confirm that centrin is recruited to peripheral aggregates and that its pericentrosomal levels increase upon centrosomal satellite clustering.

***P. 9**: "Despite prominent microtubule nucleation at the centrosomes, microtubule nucleation was not initiated at the peripheral gamma-tubulin-positive clusters." This conclusion is not supported by Fig. S3C. In the figure, it appears that there may be microtubule nucleation proportional to the amount of gamma-tubulin in the peripheral cluster as compared to the centrosome. Please provide clearer example or rephrase conclusion. In same sentence, change "gama-tubulin" to "gamma-tubulin." In same figure panel, place numbers over inset columns corresponding to boxes in large image.*

For the revised manuscript, we repeated the microtubule regrowth experiments in IMCD3 PCM1 KO^{peripheral} cells. This time, we fixed the cells at more frequent intervals after nocodazole washout (0/3/5/8/10/15 min), which allowed us to assay microtubule nucleation with higher temporal resolution. We included representative images for cells stained for microtubules, PCM1 or ninein at the indicated time points in Fig. S3C and S3D. In particular, the cells fixed 3 min and 5 min after washout convincingly showed that the gamma-tubulin positive satellite clusters did not nucleate microtubules, as shown in the new Fig. S3C. These new results confirm our initial conclusions. We note that recently published satellite proteomes identified HAUS complex components and gamma-tubulin as part of satellites and yet satellite do not function as acentrosomal MTOCS. Together with our results, we can conclude that peripheral satellite clusters or satellites lack the modifications or regulatory proteins essential for microtubule nucleation. We discuss this point with the following sentence in pg. 12:
"Thus, the concentration of gamma-tubulin at the peripheral satellites does not assign them MTOC activity, which might be due to lack of modifications or regulatory proteins required for microtubule nucleation."

P. 10. *The authors discuss "proteins enriched (>1.25 fold relative to control) or depleted (<0.75 fold relative to control)." Cutoffs of >1.25 fold and <0.75 fold do not seem very stringent for concluding that proteins are enriched or depleted. Please explain why these cutoffs were chosen and why such small changes are meaningful.*

The reviewer raises a very good point, which we should have explained in the "mass spectrometry and data analysis" section of the methods. We chose these cutoffs empirically using the following criteria that we set:

1) In Fig. 2 and S2, we used quantitative immunofluorescence to determine the proteins that are recruited to the peripheral clusters upon rapamycin treatment. In the comparative analysis of "-rapa" and "+rapa" PCM1 interactors, we determined the range above which some of these validated proteins (i.e. KIAA0753, OFD1, MIB1) are enriched (%25). For the depleted proteins, we applied the same %25 range.
2) Given that peripheral enrichment of satellites resulted in defects associated with cilium biogenesis, we also screened the comparative analysis datasets to confirm that the ranges we chose include proteins associated with primary cilium.

We now clarified in the "mass spectrometry and data analysis" part of the methods section how we chose the cutoffs for analysis (pg. 31). Finally, we acknowledge that these cutoffs can also be chosen differently in future studies to make the analysis more stringent. The Table S1 contains the raw data for mass spectrometry analysis, where the fold change for all proteins are included, which will enable use of this table as a resource in future studies.

P. 10: *"To gain insight into the specific ciliary processes that satellites regulate, we determined the PCM1 proximity interactors associated with the primary cilium in previous studies and classified them based on their functions in the ciliogenesis program in an interaction network (Fig. 3D)." Please provide citations for "previous studies." "(Fig. 3D)" here should be "(Fig. 3E)."*

- We replaced "Fig. 3D" with "Fig. 3E".
- We cited the following papers on proteomics of the primary cilium as sources of data we used to determine which PCM1 proximity interactors were associated with cilium biogenesis and functions:
Mick et al. Dev Cell 2015 (PMCID: 4662609)
Kohli et al. EMBO Reports 2017 (PCMID: 5579364)

P. 11: *"Expression of GFP-PCM1-FKBP restored the ciliogenesis defect of IMCD3 PCM1 KO cells, confirming that this fusion protein is fully functional (Fig. S5A)." Without error bars on Fig S5A, it is not clear that this conclusion is supported by the data. Please add error bars or revise statement.*

For the revised manuscript, we performed three independent rescue experiments and included the results as a bar graph in Fig. S5A. We included the associated data in pg. 15 as follows:
"Expression of GFP-PCM1-FKBP restored the ciliogenesis defect of IMCD3 PCM1 KO cells……….
(IMCD3 PCM1 KO: 56% ± 2.9, IMCD3 PCM1 KO^{peripheral}: 67.6% ± 2.1)"

Statistical analysis of the data confirmed that stable GFP-PCM1-FKBP expression restored the ciliogenesis defect of IMCD3 PCM1 KO cells. Additionally, it showed that satellite redistribution to periphery upon rapamycin addition resulted in ciliogenesis defects similar to constitutive loss of satellites.

P. 19: *"The region of interest that encompassed the centrosome was defined as a circle 3 μm^2 area centered at the centrosome in each cell." This and similar definitions currently in the Materials and Methods section are sufficiently important for understanding the experiments that they probably should be in the relevant places in the Results section.*
p.26: Complete redistribution to the cell periphery was defined by lack of centrosomal GFP-PCM1-FKBP signal in the pericentrosomal area within 3 μm^2 area centered on the centrosome.

We agree with the reviewer and included this information in the following sections as indicated below:

- Validation of satellite distribution to periphery and center (Fig. 1) (pg.8):
"To determine the targeting efficiency of endogenous PCM1, we quantified its pericentrosomal levels by measuring fluorescence signal intensities within a 3 μm^2 circle encompassing the centrosome."

- Assaying the consequences of satellite misdistribution on pericentrosomal levels of proteins (Fig. 2, Fig. S2) (pg. 10):
"To test this, we co-transfected HeLa cells with the indicated constructs and used quantitative immunofluorescence to measure the pericentrosomal levels of various known satellite proteins by measuring fluorescence signal intensities within a 3 μm^2 circle encompassing the centrosome."

P. 24: *"Images represent centrosomes in cells from the same coverslip taken with the same camera settings." As written, I am not sure this statement is needed or even relevant to the images shown. It presumably refers to the data in the scatterplots.*

We included this description as part of "Methods" section to describe the source of images used for quantitative immunofluorescence of pericentrosomal levels of select proteins. Additionally, we included this statement in the "Figure Legends for Fig. 2 / S2 /S3" to indicate that the representative images chosen for the scatter plots were taken with the same camera settings.

P. 29: *"Figure S5. Rapamycin treatment does not interfere with ciliogenesis, cilium maintenance and cilium disassembly." The title of the legend is confusing as Fig. S5D does show an experiment in which rapamycin treatment (and peripheral clustering) has an effect on ciliary maintenance. Perhaps rephrase title of figure legend.*

We agree with the reviewer that the title of Fig. S5 does not represent all the presented data and thus causes misunderstanding of our conclusions. The revised Fig. S5 now includes the following data:
(S5A) GFP-PCM1-FKBP expression restored ciliogenesis defects of IMCD3 PCM1 KO cells.
(S5B) Confirmation of GFP-PCM1-FKBP expression in IMCD3 PCM1 $KO^{peripheral}$ cells and its response to rapamycin treatment
(S5C) Quantification of ciliogenesis defects upon rapamycin treatment of IMCD3 cells expressing GFP-PCM1-FKBP and HA-Kif5b-FRB
(S5D) Effects of rapamycin treatment by itself on cilium assembly and maintenance
(S5E and F) Effect of satellite peripheral localization on cilium maintenance and disassembly

Given the content of the data related to cilium biogenesis, we changed the title of the figure to "Effects of rapamycin treatment and satellite peripheral localization on cilium assembly, maintenance and disassembly".

Editing: *The authors acknowledge Life Science Editors for editing assistance. The writing is indeed well edited up to about p. 9, and then becomes more error prone until the Discussion. Minor errors or omissions:*

We thank the reviewer for carefully reading our manuscript and finding the following errors and omissions. We corrected them as follows:

P. 8: *6th line from bottom: "(Fig. 3A)" should be "Fig. S3A."*
We corrected this mistake, pg.11 in the revised manuscript.

P. 9, *". . . how the composition of satellites upon their peripheral targeting . . ." Insert "changed" after "satellites"?*
We added "changed" to this sentence, pg.12 in the revised manuscript.

***P. 10**, line 8: I could not find a "Table 2." This probably should be "Table S2."*
We included the GO-enrichment analysis in Table S3 in the revised manuscript and correctly referenced the figure in pg. 14.

***P. 10**, last sentence: "composition peripheral satellites." Insert "of" after "composition"?*
We inserted "of" to the indicated sentence.

***P. 11:** "we used IMCD3^{peripheral} experiments for subsequent experiments at the cilia." Replace first "experiments" with "cells"? Replace "at" with "on"?*
We corrected this sentence as "we used IMCD3^{peripheral} cells for subsequent experiments at the cilia".

***P. 14:** Decimal place is indicated by period two places (31.4; 32.8) but by comma three places (32,95; 48,56; 12,94).*
We revised the numbers with two decimal places to one in order to make them consistent with how we showed that in the rest of the manuscript.

***P. 15:** "suggesting that satellites functions upstream of HDAC6." Replace "functions" with "function."*
We corrected this grammar mistake as suggested.

***P. 20:** "After on-based tryptic digest." What is "on-based"?*
We replaced "on-based" with "on-bead" to describe how tryptic digest was performed.

***P. 27,** Figure S1 legend: "excluding therosomal area in BICD2-expressing cells." "therosomal"??*
We corrected this mistake with "the centrosomal".

***P. 27,** Figure S1 legend: "F) Coexpression of GFP-PCM1-FKBP with the constitutively active HA-Kif17." "F" should be "H."*
We replaced "(F)" with "(H)".

***Figure S2B:** Add "gamma" to "-tubulin" at top left.*
We added "gamma" to indicate the type of tubulin stained.
