# Supplementary material for: Acute inhibition of centriolar satellite function and positioning reveals their functions at the primary cilium
Source: PLoS Biol. 2020 Jun 18;18(6):e3000679. doi: 10.1371/journal.pbio.3000679 (PMC7326281; doi:10.1371/journal.pbio.3000679)
Supplement: Attachment — Submitted filename: Review of PBIOLOGY-D-20-00224.docx [file pbio.3000679.s021.docx]

MS #: PBIOLOGY-D-20-00224

Title: Acute inhibition of centriolar satellite function and positioning reveals their functions at the primary cilium

This manuscript documents the development of methods based on rapamycin-induced heterodimerization of FKBP and FRB to redistribute pericentriolar satellites from their normal positions, loosely dispersed around the centrosome, either to clusters at the periphery of the cell (minus the centrosome) or to a single large cluster focused on the mother centriole. The methods are validated in great detail. The authors then use immunofluorescence microscopy to analyze how select satellite proteins change in abundance when the satellites are repositioned. Using BioID, they next determine that the satellite interactome remains relatively unaltered when the satellites are redistributed to peripheral clusters. The authors then carry out studies to determine how ciliary assembly, maintenance, and disassembly are affected by repositioning of the satellites to the cell periphery. The results provide the first direct evidence that proximity of the satellites to the cilium’s base is important for primary cilium maintenance and disassembly. In addition to extending the general importance of cell organelle positioning for function of the cell organelle, the results raise interesting questions for future research. In general, the manuscript is scholarly, well written, and scientifically sound; it is appropriate for publication in PLOS Biology. However, it would be improved if the following issues were addressed prior to publication.

P. 4: The major section that begins here is called “Results and Discussion.” There also is a “Discussion” section that begins on p. 14. The section beginning on p. 4 probably should be simply “Results.”

P. 5: The phrase “co-expressing the FKBP-fusion of satellites as the cargo of interest” is awkward and unclear.

P. 5: HA-BICD2 expression by itself caused satellite dispersal, presumably as a result of its previously documented dominant-negative effect on dynein-dynactin function. Here or later, briefly explain why addition of rapamycin to these cells nevertheless results in clustering of satellites at the centrosome, presumably due to dynein moving on microtubules to their minus ends.

P. 7: Clustering of satellites is dependent on microtubules, but the clusters do not disperse when microtubules are depolymerized. Briefly discuss the likely reason that the clusters remain intact in the absence of microtubules.

P. 8: “except for centrin.” I could not find data for centrin. Add “data not shown”?

P. 9: “Despite prominent microtubule nucleation at the centrosomes, microtubule nucleation was not initiated at the peripheral gama-tubulin-positive clusters.” This conclusion is not supported by Fig. S3C. In the figure, it appears that there may be microtubule nucleation proportional to the amount of gamma-tubulin in the peripheral cluster as compared to the centrosome. Please provide clearer example or rephrase conclusion. In same sentence, change “gama-tubulin” to “gamma-tubulin.” In same figure panel, place numbers over inset columns corresponding to boxes in large image.

P. 10. The authors discuss “proteins enriched (>1.25 fold relative to control) or depleted (<0.75 fold relative to control).” Cutoffs of >1.25 fold and <0.75 fold do not seem very stringent for concluding that proteins are enriched or depleted. Please explain why these cutoffs were chosen and why such small changes are meaningful.

P. 10: “To gain insight into the specific ciliary processes that satellites regulate, we determined the PCM1 proximity interactors associated with the primary cilium in previous studies and classified them based on their functions in the ciliogenesis program in an interaction network (Fig. 3D).” Please provide citations for “previous studies.” “(Fig. 3D)” here should be “(Fig. 3E).”

P. 11: “Expression of GFP-PCM1-FKBP restored the ciliogenesis defect of IMCD3 PCM1 KO cells, confirming that this fusion protein is fully functional (Fig. S5A).” Without error bars on Fig S5A, it is not clear that this conclusion is supported by the data. Please add error bars or revise statement.

P. 19: “The region of interest that encompassed the centrosome was defined as a circle 3 μm^2^ area centered at the centrosome in each cell.” This and similar definitions currently in the Materials and Methods section are sufficiently important for understanding the experiments that they probably should be in the relevant places in the Results section.

P. 24: “Images represent centrosomes in cells from the same coverslip taken with the same camera settings.” As written, I am not sure this statement is needed or even relevant to the images shown. It presumably refers to the data in the scatterplots.

P. 29: “Figure S5. Rapamycin treatment does not interfere with ciliogenesis, cilium maintenance and cilium disassembly.” The title of the legend is confusing as Fig. S5D does show an experiment in which rapamycin treatment (and peripheral clustering) has an effect on ciliary maintenance. Perhaps rephrase title of figure legend.

The authors acknowledge Life Science Editors for editing assistance. The writing is indeed well edited up to about p. 9, and then becomes more error prone until the Discussion. Minor errors or omissions:

P. 8: 6^th^ line from bottom: “(Fig. 3A)” should be “Fig. S3A.”

P. 9, “. . . how the composition of satellites upon their peripheral targeting . . .” Insert “changed” after “satellites”?

P. 10, line 8: I could not find a “Table 2.” This probably should be “Table S2.”

P. 10, last sentence: “composition peripheral satellites.” Insert “of” after “composition”?

P. 11: “we used IMCD3^peripheral^ experiments for subsequent experiments at the cilia.” Replace first “experiments” with “cells”? Replace “at” with “on”?

P. 14: Decimal place is indicated by period two places (31.4; 32.8) but by comma three places (32,95; 48,56; 12,94).

P. 15: “suggesting that satellites functions upstream of HDAC6.” Replace “functions” with “function.”

P. 20: “After on-based tryptic digest.” What is “on-based”?

P. 27, Figure S1 legend: “excluding therosomal area in BICD2-expressing cells.” “therosomal”??

P. 27, Figure S1 legend: “F) Coexpression of GFP-PCM1-FKBP with the constitutively active HA-Kif17.” “F” should be “H.”

Figure S2B: Add “gamma” to “-tubulin” at top left.
